# Vector competence of *Anopheles stephensi* for O’nyong-nyong virus: a risk for global virus spread

**DOI:** 10.1186/s13071-023-05725-0

**Published:** 2023-04-17

**Authors:** Maud Mutsaers, Cecilia Springer Engdahl, Lukas Wilkman, Clas Ahlm, Magnus Evander, Olivia Wesula Lwande

**Affiliations:** 1 Department of Clinical Microbiology, Umeå University, 901 85 Umeå, Sweden; 2 Umeå Centre for Microbial Research (UCMR), Umeå University, 90187 Umeå, Sweden

**Keywords:** *O’nyong-nyong virus*, *Anopheles stephensi*, Vector competence, *Alphavirus*, Arthritis

## Abstract

**Background:**

O’nyong-nyong virus (ONNV) is a mosquito-borne alphavirus causing sporadic outbreaks of febrile illness with rash and polyarthralgia. Up to now, ONNV has been restricted to Africa and only two competent vectors have been found, *Anopheles gambiae* and *An. funestus*, which are also known malaria vectors. With globalization and invasive mosquito species migrating to ONNV endemic areas, there is a possible risk of introduction of the virus to other countries and continents. *Anopheles* *stephensi,* is closely related to *An. gambiae* and one of the invasive mosquito species of Asian origin that is now present in the Horn of Africa and spreading further east. We hypothesize that *An. stephensi*, a known primary urban malaria vector, may also serve as a new possible vector for ONNV.

**Methods:**

One-week-old female adult *An.* *stephensi* were exposed to ONNV-infected blood, and the vector competence for ONNV, i.e. infection rates (IRs), dissemination rates (DRs), transmission rates (TRs), dissemination efficiency (DEs) and transmission efficiency (TEs), were evaluated. Infection (IRs), dissemination efficiency (DEs) and transmission efficiency (TEs) were determined. Detection of ONNV RNA was analysed by RT-qPCR in the thorax and abdomen, head, wings, legs and saliva of the infected mosquitoes at four different time points, day 7, 14, 21 and 28 after blood meal. Infectious virus in saliva was assessed by infection of Vero B4 cells.

**Results:**

The mean mortality across all sampling times was 27.3% (95 confidence interval [CI] 14.7–44.2%). The mean rate of infection across all sampling periods was 89.5% (95% CI 70.6–95.9). The mean dissemination rate across sampling intervals was 43.4% (95% CI 24.3–64.2%). The mean TR and TE across all mosquito sampling time intervals were 65.3 (95% CI 28.6–93.5) and 74.6 (95% CI 52.1–89.4). The IR was 100%, 79.3%, 78.6% and 100% respectively at 7, 14, 21 and 28 dpi. The DR was the highest at 7 dpi with 76.0%, followed by 28 dpi at 57.1%, 21 dpi at 27.3% and 14 dpi at the lowest DR of 13.04%. DE was 76%, 13.8%, 25%, 57.1% and TR was 79%, 50%, 57.1% and 75% at 7, 14, 21 and 28 dpi respectively. The TE was the highest at 28 dpi, with a proportion of 85.7%. For 7, 14 and 21 dpi the transmission efficiency was 72.0%, 65.5% and 75.0% respectively.

**Conclusion:**

*Anopheles stephensi* is a competent vector for ONNV and being an invasive species spreading to different parts of the world will likely spread the virus to other regions.

**Graphical Abstract:**

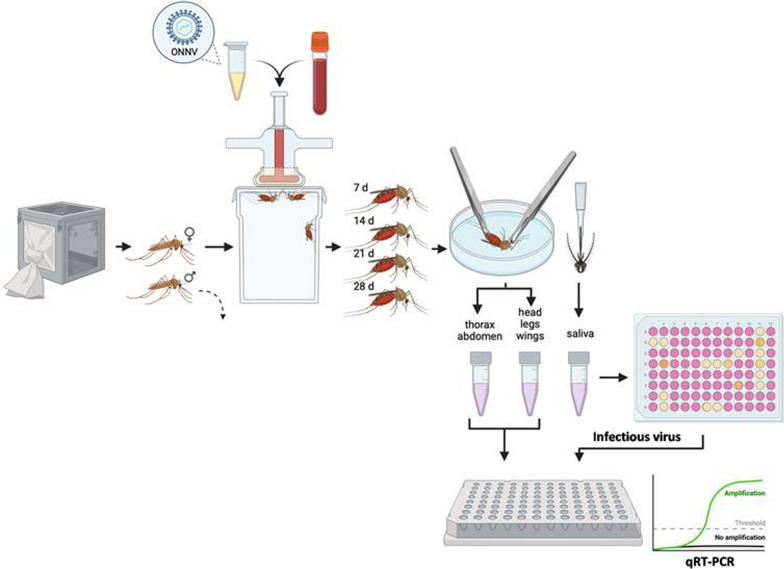

## Background

O’nyong-nyong virus (ONNV) is a mosquito-borne *alphavirus* that belongs to the *Togaviridae* family. ONNV is infection in humans is characterized by fever, maculopapular skin rash, myalgia, incapacitating polyarthralgia and extended lymphadenopathy [1, 2]. This results in high morbidity in humans hence raising concern about public health [3].

Multiple outbreaks of ONNV have occurred in the previous century and a more recent epidemic in 2015 in East Africa [4]. During the initial outbreak that occurred 1959–1962, more than 2 million people were affected in Uganda, Kenya, Tanzania, Senegal, Cameroon, Central African Republic, Democratic Republic of Congo, Malawi and Mozambique [5–7]. So far, three strains of ONNV have been recognized: Gulu (Uganda, 1959), SG650 (Uganda, 1996) and Igbo Ora virus (Nigeria, 1966) [8–12], and all are restricted to Africa. However, there is a concern that ONNV may spread to other continents [3]. In 2013, a 60-year-old woman living in Germany was confirmed positive for ONNV after vacationing in Kenya near Lake Victoria where outbreaks had previously been reported [13]. What is limiting the spread of ONNV is likely the vector. There are only two known vectors of ONNV, *Anopheles* *funestus* and *An.* *gambiae* [3, 11], and these vectors have not been found outside of tropical Africa [6].

Also, little is known about the enzootic cycle of ONNV. Besides humans, no other vertebrate reservoirs have been discovered yet [2, 14]; current serological evidence shows that ONNV is mostly circulating in sub-Saharan Africa.

*Anopheles stephensi* is originally endemic to South-East Asia and a large part of the Arabian Peninsula and is also a known vector for *Plasmodium* *falciparum* and *P. vivax* [15]. There have been reports of *An. stephensi* in countries within the Horn of Africa, including Djibouti and Ethiopia. It has also spread further south and can now be found in Sudan in sub-Saharan Africa [15]. The prognosis is that *An.* *stephensi* will spread to densely populated urban areas in the malaria-endemic zone. Several of these cities are in countries where ONNV is established, such as Nairobi and Mombasa in Kenya and Kampala in Uganda [4, 5, 16].

In recent years, ONNV research has grown with studies mostly focusing on the known vectors *An.* *funestus* and *An.* *gambiae*. Research regarding the mosquitoes’ immune responses and mechanisms towards ONNV infection and genetic modulation after infection have been extensively performed [1, 17–22]. Interestingly, a study on the resistance of genetically modified *An. stephensi* to *P. falciparum* infection included ONNV as a control, and the authors reported ONNV in *An. stephensi* midgut tissue 5 days after infection [23], but no further study on the potential of *An. stephensi* as a vector was performed. We hypothesize, based on these findings and the fact that *An. stephensi* is closely related to *An.* *gambiae*, that *An. stephensi* may be susceptible to ONNV. Therefore, this study aimed to determine the vector competence of the invasive urban malaria mosquito *An.* *stephensi* for ONNV.

## Methods

### Mosquito rearing

The *An.* *stephensi* mosquito colony, strain Sind-Kasur 500 (SDA-500), origin Pakistan, was kindly provided as mosquitoes (day 4 post-eclosion) from the Oliver Billker Insectary, Molecular Infection Medicine Sweden, Umeå University. The colony was originally obtained from Radboud University, Nijmegen, The Netherlands. Approximately 400 adult mosquitoes were reared and maintained in a climate chamber (Memmert GmbH & Co. KG, Konstantklima-Kammer, HPP410, Schwabach, Germany) at 28 °C ± 1 °C, 12:12 h light:dark diurnal cycle at 80% relative humidity in cages. Mosquitoes were provided with 10% sucrose ad libitum for maintenance.

### Virus strain

ONNV SG650, which was first isolated from human serum in Uganda in 1996, was used in the study (GenBank AF079456.1). The virus was provided by the Division of Vector-Borne Diseases (DVBD), Centers for Disease Control and Prevention (CDC), Fort Collins, Colorado, USA. The virus stock was obtained after two passages on Vero B4 cells in Dulbecco’s minimum essential medium (DMEM; Gibco^®^) supplemented with 2% foetal bovine serum (FBS; GE Healthcare Life Sciences, Cramlington, UK), 2% HEPES, 2% penicillin-streptomycin (PEST; GE Healthcare Life Sciences, South Logan, UT) and 2% l-glutamine (Gibco). The supernatant was stored in aliquots at − 80 °C until use for mosquito infection.

### Standard curve

**Fig. 1:**
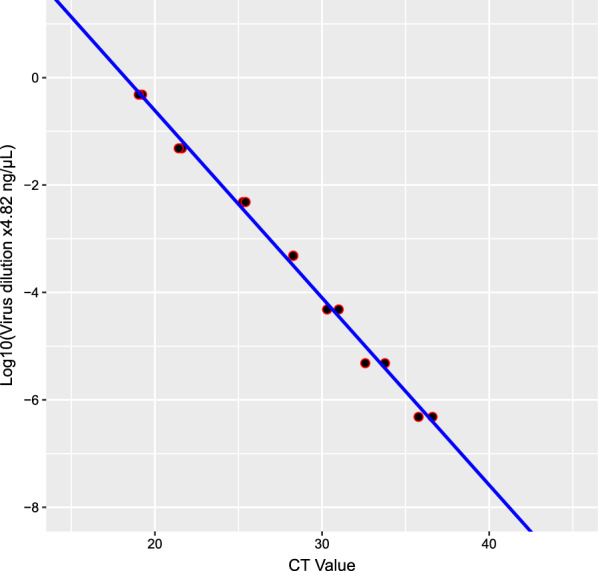
Virus dilution curve and its relationship with CT (cycle threshold)

The ONNV RNA concentration determined by Qubit 4 Fluorometer (Invitrogen) as 4.82 ng/µl was made into seven tenfold serial dilutions prepared in duplicate and quantified by qRT-PCR. The CT values obtained were used to generate a standard curve (Fig. 1).

### Vector competence of *An. stephensi* for ONNV

This was an experimental research study conducted in an Arthropod Containment Level-2 (ACL-2) Laboratory located at the Department of Clinical Microbiology, Umeå University, Umeå, Sweden. To assess the vector competence, female adult *An.* *stephensi* mosquitoes were exposed to ONNV-infected blood and followed at weekly intervals for up to 28 days after the blood meal with analyses of the infection rate, transmission rate and transmission efficiency.

One-week-old adult mosquitoes (*n* = 400) were starved for 24 h before being allowed to feed on ONNV infectious blood meals via a Hemotek artificial membrane feeder, (Hemotek Ltd, Blackburn, UK). The human blood (anonymous blood from the Blood Centre at the University Hospital of Umeå, Sweden) contained a final titre of 2 × 10^8^ plaque-forming units/ml ONNV, which was limited to the available stock concentration provided by DVBD, CDC. The ratio of human blood to virus cell culture media (DMEM) was 1:1. We used fresh blood so that it was easy to see the reddish colour of the distended abdomen to differentiate between engorged and non-engorged mosquitoes. The size of the abdomen was used to differentiate between fully and partially engorged. Partially engorged mosquitoes are the abdomen intermediate between non- and fully engorged mosquitoes.

Mosquitoes were allowed to feed for approximately 1 h, and then partially and non-blood-fed females and males were discarded. An aliquot of the blood meal was archived for virus blood titre verification. Two engorged mosquitoes were also archived at − 80 °C immediately after exposure to the ONNV infectious blood meal. The remaining engorged mosquitoes (each containing 33) were separated into four groups containing the same number of mosquitoes representing four different time points. The infected mosquitoes were fed with 10% sucrose ad libitum and maintained under standard rearing conditions until dissection.

ONNV infection and dissemination were assessed at 7, 14, 21 and 28 days post-infection (dpi). Female mosquitoes were knocked out by placing them at − 20 °C for 30–60 s. While knocked out, the mosquitoes were dissected on ice by removing the legs and the wings, which were placed in a 2-ml tube with 350 µl DMEM (Gibco^®^) and three stainless steel beads (NinoLab AB, Stockholm, Sweden). The proboscis of each now immobilised mosquito was then positioned in a 20-µl tapered pipette tip containing 5 µl of a 1:1 solution of 50% sucrose and FBS (GE Healthcare Life Sciences, Cramlington, UK) to induce salivation. After 30 min, each pipette tip content was expelled into 45 µl DMEM, and the head was separated from the thorax and abdomen. The head was placed in the same tube as the wings and legs. The thorax and abdomen were placed in a separate tube containing 350 µl DMEM (Gibco^®^) and three beads. The mosquito’s dissected parts and saliva were stored at – 80 °C until tested for ONNV presence.

### Analysis of dissected mosquito parts and saliva

To analyse for ONNV RNA, the mosquito body parts were thawed at room temperature and homogenised in a FastPrep-24 homogeniser (Mpbiomedicals, USA) at 40 m/s for 20 s. The mosquito’s thorax and abdomen and the combined legs/wings/head samples were tested for ONNV RNA presence through RNA extraction using the QIAamp Viral RNA kit (Qiagen, Valencia, CA, USA) according to manufacturer’s protocol followed by q-RT-PCR. The mosquito saliva was tested for ONNV presence by cytopathogenic effect (CPE) on cell cultures followed by RNA extraction and qPCR. Vero B4 cells with a seeding density of 20,000 cells/well were seeded in a 96-well plate. When the wells had reached confluency, the cells were infected with 20 µl of saliva sample. The inoculated saliva samples were manually monitored for CPE daily for a week, and wells that showed CPE were harvested; RNA was extracted and q-RT-PCR performed. For the qPCR (Fisher Biosystems) we used Biosystems qPCRBIO Probe 1-step Go Lo-ROX kit and the following protocol: 1 cycle at 45 °C for 10 min, 1 cycle at 95 °C for 5 min and 40 cycles at 60 °C using primers targeting the ONNV Envelope genes E1 and E2 [24]. The specificity and sensitivity are shown elsewhere [24]. The probe was designed in close proximity to the forward and reverse primer and did not overlap with a primer binding site on the same site [24].

### Data analysis

To establish vector competence for ONNV, we determined mosquito infection rates (IRs), virus dissemination rates (DRs) and transmission rates (TRs), virus dissemination efficiencies (DEs) and transmission efficiencies (TEs) at different time points post-mosquito infection. IR(s) was calculated as the proportion of mosquitoes with infected bodies (positive thorax and abdomen) among tested mosquitos. DR(s) was calculated as the proportion of mosquitoes with the infected legs wings and head among those having an infected body (thorax and abdomen). TR is the proportion of mosquitoes with infectious saliva among mosquitoes with disseminated infection (ONNV-positive head, legs and wings). DE and TE refer to the proportion of mosquitoes with infectious viral particles in the legs or in the saliva, respectively, among all tested mosquitos [25–27]. The binomial function was used to determine the confidence interval of a proportion using the Clopper-Pearson method for confidence intervals. To test whether variation in IR, DR, TR, DE, TE and mosquito mortality varied across time points, the chi-square test of proportions was used and the probability values were generated following a simulation with default software parameters. All statistical analyses were performed using the R software for statistical computing [28].

## Results

For each of the time points (7, 14, 21 and 28 days post-infection), 33 mosquitoes each that were offered an infectious blood meal with ONNV clearly displayed a blood meal in the abdomen. In addition, a positive control consisting of two mosquitoes of the same batch offered an infectious blood meal and harvested directly after feeding tested positive for ONNV by qRTPCR test and five mosquitos that were unfed and used as a negative control also turned negative for ONNV by qRTPCR test.

The mean ± (SD) CT values for mosquito infection (ONNV-positive thorax and abdomen), dissemination (ONNV positive head, legs and wings) and transmission (ONNV positive saliva) were lowest for saliva (26.55 ± 7.58), moderate for abdomen and thorax (32.66 ± 4.027) but highest (34.58 ± 5.36) for head, legs and wings (Table 1). The standard curve indicates a relatively low virus concentration for infected mosquitos (CT 33), which was approximately 4.82 × 10^–5^ ng/µl (Fig. 1) compared to the cut-off (CT 40) at nearly 4.82 × 10^–8^ ng/µl dilution.

**Table1:** Mean ± (SD) CT values for positive infections, dissemination and transmission at weekly intervals

Days post-infection (dpi)	Abdomen and thorax	Wings, legs and head	Saliva
7	30.73 ± 4.62	35.56 ± 4.41	25.14 ± 1.03
14	32.01 ± 3.45	33.46 ± 6.05	29.47 ± 2.02
21	35.68 ± 2.89	31.27 ± 8.40	24.19 ± 1.98
28	32.40 ± 3.90	35.73 ± 4.42	28.88 ± 1.39
Mean	32.66 ± 4.027	34.58 ± 5.36	26.55 ± 7.58

**Fig. 2:**
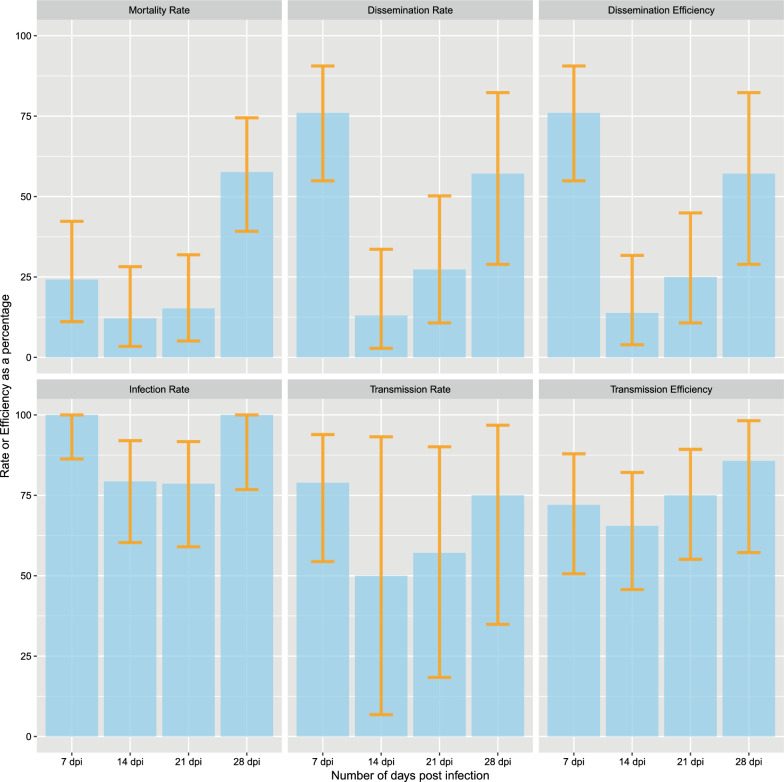
Mosquito competence parameters including, mosquito mortality, virus infection, dissemination and transmission. Error bars showing 95% confidence interval are included

There was a statistically significant difference in mosquito mortality at different time intervals post-infection (Table 2). The mean mortality across all sampling times was 27.3% (95 confidence interval (CI): 14.7–44.2%). Mortality at 7 dpi was eight (24.2%); at 14 dpi this was four (12.12%) and five (15.2%) at 21 dpi. The difference in mortality rate across time periods post-infection was driven by a high mortality at 28 dpi, namely 19 (57.58%) mosquitoes (Table 2, Fig. 2).

**Table 2:** Infection, dissemination, transmission and mortality parameters and (95% CI) with lower and upper confidence intervals

Measure	7 dpi		14 dpi		21 dpi		28 dpi		Probability	
	Value (95% LCI-UCL)	*N*	Value (95% LCI-UCL)	*N*	Value (95% LCI-UCL)	*N*	Value (95% LCI-UCL)	*N*	*χ*2	*P*. value
Mortality rate	24.24 (11.09–42.26)	33	12.12 (3.40–28.20)	33	15.15 (5.11–31.90)	33	57.58 (39.21–74.52)	33	21.69	0.0005
Infection rate	100.0 (86.28–100.0)	25	79.31 (60.28–92.01)	29	78.57 (59.05–91.70)	28	100.0 (76.84–100.0)	14	8.34	0.0305
Dissemination rate	76.00 (54.87–90.64)	25	13.04 (2.78–33.59)	23	27.27 (10.73–50.22)	22	57.14 (28.86–82.34)	14	22.9	0.0005
Dissemination efficiency	76.00 (54.87–90.64)	25	13.79 (3.89–31.66)	29	25.00 (10.69–44.87)	28	57.14 (28.86–82.34)	14	26.22	0.0005
Transmission rate	78.95 (54.43–93.95)	19	50.00 (6.76–93.24)	4	57.14 (18.41–90.10)	7	75.00 (34.91–96.81)	8	2.16	0.5742
Transmission efficiency	72.00 (50.61–87.93)	25	65.52 (45.67–82.06)	29	75.00 (55.13–89.31)	28	85.71 (57.19–98.22)	14	2.04	0.5957

The infection rates were variable between days post-infection and these differences were statistically significant (Table 2). The mean rate of infection across all sampling periods was 89.5% (95% CI 70.6–95.9). The difference in infection rates was higher and most different at 7 dpi at 100% (95% CI 86.28–100.00). The infection rates were similar (within statistical error) at 14 dpi 79.31% (95% CI 60.28–92.01), 21 dpi 78.57% (95% CI 59.05–91.70) and 28 dpi 100% (95% CI 76.84–100.00) (Table 2, Fig. 2).

The mean dissemination rate across sampling intervals was 43.4% (95% CI: 24.3–64.2%). The dissemination rates were statistically different across post-infection time periods (Table 2). DR was the highest at 7 dpi with 76.0% followed by 28 dpi at 57.14%. It was lowest at 14 dpi at 13.04% followed by 21 dpi at 27.274% (Table 2, Fig. 2). The mean DE was 43.0% (95% CI 24.6–62.4). Dissemination efficiencies (DEs) across sampling time intervals followed a similar pattern to DRs (Table 2, Fig. 2).

The mean TR and TE across all mosquito sampling time intervals were 65.3 (95% CI: 28.6–93.5) and 74.6 (95% CI 52.1–89.4). Transmission rates and transmission efficacies of ONNV in *An. stephensi* varied across all time points but the differences were not statistically significant for both measures (Table 2, Fig. 2). The transmission rate varied from the lowest rate at 7 dpi, which was 78.95%, to 14 dpi, which was 50.00%. The transmission efficiency was the highest at 28 dpi, with 85.71%, and lowest at 14 dpi, which was 65.52% (Table 2, Fig. 2).

Vector competence parameter values for IRs, DRs and DE appear to follow a U-shape pattern over time post-infection. The highest values of these parameters were recorded at 7 dpi and then dramatically dropped to the lowest at 14 dpi before gradually rising to nearly high levels at 28 dpi, similar to those observed at 7 dpi. Mortality rate followed a similar pattern but was more like a J-pattern.

## Discussion

In this study, we experimentally demonstrated that *An. stephensi* is susceptible to infection by ONNV with infection rates ranging from ~ 79% to 100%. The infection rates we observed were comparable to infection rates recorded for competent mosquito vectors in other studies [29–31]. For example, the infection rates were 100% and 82% for *Aedes albopictus* an *Anopheles quadrimaculatus* respectively for Mayaro virus [30]. In another study, the infection rates for *Ae. albopictus*-LBV, *Ae. albopictus* FCV and *Ae. aegypti*-FCV, for the African strain of Zika virus, DAK84, were 73.3–86.7, 83.3–90.0 and 60.0–93.3 respectively [25].

This study also revealed that experimentally infected *An. stephensi* mosquito with ONNV disseminated the virus at rates (13–76%) and efficiencies (14–76%) comparable to those of other competent mosquito vectors. For example, *Ae.* *albopictus* and *An. quadrimaculatus* had dissemination rates of 95.6% and 61.0% and dissemination efficiencies of 95.6% and 50.0% respectively for Mayaro virus. The dissemination rates of West Nile virus in *Ae. albopictus * and *Culex pipiens* were 75% and 59% respectively [29]. In this study, unlike previous ones, the dissemination rate was initially high at 7 dpi and then dropped to its lowest at 14 dpi before gradually increasing to attain rates closer to those observed at 7 dpi. The high IRs, DRs and DEs recorded for ONNV in *An. stephensi* suggest the capability for this virus to bypass both the midgut and salivary gland barriers in this mosquito. Following a viraemic blood meal by mosquitoes, virus enters the midgut along with the blood, infects and replicates in midgut epithelial cells and then escapes to the haemocoel, from where it disseminates to various organs including legs, wings, head and salivary glands. The midgut and salivary glands act as anatomical barriers to virus infection and escape [32]. The changes in virus dissemination also mirror viral infection rates over time following infection and suggest a dynamic interaction between the virus and the mosquito immunity. Initially, the virus escapes immunity and disseminates to other parts of the body a week post-infection. By the second week the mosquito immune response may have cleared infection in some individuals before the virus gradually escapes the immune response again to attain a higher degree of infection by 28 dpi.

The present study revealed a high infection, dissemination and transmission rate as early as 7 days post-infection. A possible explanation could be that ONNV is an alphavirus and upon entry in the midgut it was able to replicate in the midgut within a short time, hence spreading to other parts of the mosquito. Although vector competence is influenced by many variables, such as initial virus titres or on the virus strain, we only tested the vector competence of *An. stephensi* for ONNV using the SG650 strain, which has been previously demonstrated to replicate efficiently in both *An. gambiae* and *Ae. aegypti*; the strain has been reported to maintain its natural phenotype and has not undergone several passages compared to the Gulu and Igbo Ora strains [33]. Evidence of ONNV dissemination in *An. gambiae* has been reported to occur as early as 3 days post-infection [34], and ONNV infection in *An. stephensi* seemed to result in a relatively fast replication as well. The mortality rate was highest at 28 days post-infection. Preliminary findings from our laboratory (unpublished) on *An. stephensi* longevity is that it is dependent on temperature, with a decrease in survival beyond 21 days at 28 °C. In addition, for malaria, temperature has a great influence on the transmission capacity of *An. stephensi* depending on the *Plasmodium* species, for example for *P. falciparum* it has a breadth temperature range of 15.3–37.2 °C whereas for *P. vivax* it is between 15.7 °C and 32.5 °C [35].

The transmission rate and transmission efficiency observed in this study were similar to observations made by other studies on competent vectors suggesting that *An. stephensi* is a competent vector for ONNV transmission. However, the viral transmission rate and efficiency were higher than the dissemination rate, yet in other studies the transmission rates and efficiency are lower than viral dissemination rates. There are two plausible explanations for the observed pattern. The pattern is perhaps due to amplification of the virus in cell culture. A possible explanation for the lower dissemination rate could be that *An. stephensi* had an immune response, that after a certain time the virus was restricted to the thorax, abdomen and salivary glands, or that the virus was present in wings, legs and head, but in too low concentrations to be measured with qPCR. Second, qPCR was performed only on samples from Vero B4 cells that showed CPE, which probably resulted in amplification of the virus before the qPCR. We also presume that the assay may not have been sensitive enough when the virus RNA concentration was low.

Apart from ONNV, the *Anopheles* species are known to transmit other viruses. For example *An.* quadrimaculatus has been shown to transmit Mayaro virus and Cache Valley virus at 7 and 14 dpi respectively in the USA [30, 36]. Other viruses such as chikungunya virus and Jamestown Canyon virus have been detected from *Anopheles* [37, 38], hence increasing the potential risk of *Anopheles* mosquitoes including *An. stephensi* in the transmission of arboviruses.

This study confirmed that *An. stephensi* is a competent vector for ONNV. We believe that the vector is capable of transmitting the virus in ONNV endemic areas, introducing the virus to new areas within the African continent and spreading to other continents. In addition, the invasive nature of the vector may increase the risk of local transmission of ONNV leading to disease outbreaks. Originally the vector was restricted to South Asia and the Middle East including the Arabian Peninsula [39]. However, reports of the vector spread and persistence in Africa have been made in Djibouti, Ethiopia, Somalia, Sudan and the rest of the Horn of Africa [40–42]. However, the ONNV enzootic cycle remains unknowns; the virus is known to primarily circulate amongst humans via *Anopheles* spp. mosquitoes, which may increase the likelihood of the adaptability of the vector in sustaining the virus transmission in urban areas where humans are abundant [11]. This is evident in the case of the closely related chikungunya virus (CHIKV) whose global expansion and re-emergence were linked to the viral adaptation to a new mosquito vector, *Ae. albopictus*, which facilitated the transmission of the virus to areas which were not previously colonised by the native vector *Ae. aegypti*. It has been proven that a single mutation at position 226 of envelope gene 1 (E1) in CHIKV, where there was an interchange of alanine with valine, led to increased virus infection, dissemination and transmission by *Ae. albopictus* [43, 44].

## Conclusions

This study provides preliminary findings indicating that *An. stephensi* is a competent vector for ONNV when maintained at 28 °C, with the peak for transmission efficiency at 28 dpi and in single virus concentration in blood of 2 × 10^8^ plaque-forming units/ml diluted by 50%. The findings also imply the potential risk of introduction of *An. stephensi* to ONNV-endemic areas and introduction of ONNV to other African countries and continents. There is a need to investigate the effects of different viral concentrations and strains and of mosquito-raising conditions such as temperature and humidity on the competence of *An. stephensi* for ONNV.

## Data Availability

All data that this paper relies upon is presented within this article.

## Abbreviations

ACL: Arthropod containment level 2
CDC: Centres for Disease Control and Prevention
CHIKV: Chikungunya virus
CPE: Cytopathic effect
CT: Cycle threshold value
DMEM: Dulbecco’s minimum essential medium
DVBD: Division of Vector-borne and Zoonotic Diseases
FBS: Fetal bovine serum
ONNV: O’nyong-nyong virus
PEST: Penicillin and streptomycin
RNA: Ribonucleic acid
q-RT-PCR: Quantitative reverse transcriptase polymerase chain reaction
